# Reliability and Validity of the Short-Form 12 Item Version 2 (SF−12v2) Health-Related Quality of Life Survey and Disutilities Associated with Relevant Conditions in the U.S. Older Adult Population

**DOI:** 10.3390/jcm9030661

**Published:** 2020-02-29

**Authors:** Chintal H. Shah, Joshua D. Brown

**Affiliations:** 1 Department of Pharmaceutical Health Services Research, University of Maryland School of Pharmacy, Baltimore, MD 21201, USA; chintalshah@umaryland.edu; 2 Center for Drug Evaluation and Safety, Department of Pharmaceutical Outcomes and Policy, University of Florida College of Pharmacy, Gainesville, FL 32610, USA

**Keywords:** older adults, SF−12v2, Medial Expenditure Panel Survey, utility, disutility, quality of life, health-related quality of life, reliability, validity, psychometric properties

## Abstract

This study aimed to validate the Short-Form 12-Item Survey—version 2 (SF−12v2) in an older (≥65 years old) US population as well as estimate disutilities associated with relevant conditions, using data from the Medical Expenditure Panel Survey longitudinal panel (2014–2015). The physical component summary (PCS) and mental component summary (MCS) scores were examined for reliability (internal consistency, test-retest), construct validity (convergent and discriminant, structural), and criterion validity (concurrent and predictive). The study sample consisted of 1040 older adults with a mean age of 74.09 years (standard deviation: 6.19) PCS and MCS demonstrated high internal consistency (Cronbach’s alpha—PCS: 0.87, MCS: 0.86) and good and moderate test-retest validity, respectively (intraclass correlation coefficient: PCS:0.79, MCS:0.59)). The questionnaire demonstrated sufficient convergent and discriminant ability. Confirmatory factor analysis showed adequate fit with the theoretical model and structural validity (goodness of fit = 0.9588). Concurrent criterion validity and predictive criterion validity were demonstrated. Activity limitations, functional limitations, arthritis, coronary heart disease, diabetes, myocardial infarction, stroke, angina, and high blood pressure were associated with disutilities of 0.18, 0.15, 0.06, 0.07, 0.07, 0.06, 0.09, 0.06, and 0.08, respectively, and demonstrated the responsiveness of the instrument to these conditions. The SF−12v2 is a valid and reliable instrument in an older US population.

## 1. Introduction

In the United States in 2018, 16% of the population was aged 65 years or older, which is a 3.2% increase from the previous year [1]. Since 2010, this age group has increased by 30.2%, with the aging of the Baby Boomers contributing to this rise [1]. Quality of life is widely used as a significant health outcome indicator [2]. When used in a healthcare and disease context, quality of life is referred to as health-related quality of life, which is a multidimensional concept that entails the domains related to mental, physical, social, and emotional functioning [3]. Health utilities enable us to place health-related quality of life on a scale, where 1 implies perfect health and 0 implies death [4,5]. There are a variety of instruments available to measure and quantify quality of life and it is important that there is sufficient evidence demonstrating the reliability and validity of the chosen instrument in order for the results to be credible [6,7]. 

The Medical Outcomes Study Short-Form 12-Item Health Status Survey—version 2 (SF−12v2) is one such instrument, and it takes less than two minutes to administer [8,9]. The Medical Expenditure Panel Survey includes the SF−12v2 instrument [10]. Previous studies have used the SF−12v2 instrument to quantify health related quality of life in an older population [11,12,13,14,15] and although this instrument has been validated in other groups [16,17,18,19,20,21], there is a need to validate this instrument among older adults. This study aims to evaluate the psychometric properties of the SF−12v2 among older adults using data from the Medical Expenditure Panel Survey and classical testing methods. 

## 2. Research Design and Methods 

### 2.1. Data Source and Study Cohort 

This study utilized data from the Medical Expenditure Panel Survey, which was provided by the Agency for Healthcare Research and Quality and is publicly available [10]. It consists of a large set of survey data that has been collected since 1996, from families and individuals, their medical providers, and employers across the United States. These data consist of a rotating panel of individuals and each panel is followed for a period of two years. The household component of this data was used. The household component is based on answers provided to questionnaires by individual household members and their medical providers. The household component collects data on demographic characteristics, health conditions, healthcare use, health status (mental and physical), access to care, insurance status, income, employment, and payment information for each individual in a household. Specifically, the data files we utilized were the longitudinal panel 19 data file (corresponding to 2014 and 2015) and the 2014 and 2015 medical condition files. 

**Figure 1:**
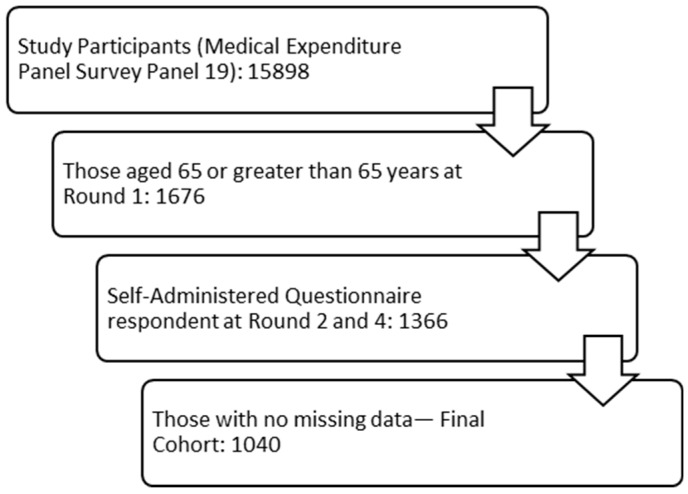
Figure depicting the sample selection process.

The study cohort consisted of all respondents who were aged 65 years or older at baseline at the beginning of the survey (Round 1) [22,23]. Among these people, only those who responded to the self-administered questionnaire portion of the survey for Rounds 2 and 4 and had no missing data for the variables of interest were retained in the final study cohort. The sample selection process has been depicted in Figure 1.

### 2.2. Demographic Information 

The baseline characteristics of the individuals were measured at Round 1 or Round 2, if that was the first time the measurement was made, as is the case with variables based on the self-administered questionnaire portion. The characteristics examined included marital status, census region, insurance coverage, race, sex, limitations in work, housework or school, functional limitations, age, physical component summary (PCS) score, mental component summary (MCS) score, health-related quality of life comorbidity indices (HRQoL-CI), and Short-Form Six-Dimension (SF−6D) scores. Marital status was recategorized to consist of three categories: never married, widowed/divorced/separated, and currently married. Those who had a change in category in the round were considered to be a member of the new category (for example, someone who was “married in round” was considered to be currently married). Race was also recategorized to create three categories: white, black, and other. 

### 2.3. Measures of Interest

#### 2.3.1. Study Short-Form 12-Item Health Status Survey—Version 2

The SF−12v2 is a concise version of the Study Short-Form 36-Item Health Status Survey—version 2 (SF−36v2) and uses only 12 questions to measure functional health and well-being from a patient-reported perspective [8,24]. It covers the same eight domains of health as the SF−36v2, which are: general health, physical functioning, role functioning (physical), bodily pain, vitality, role functioning (emotional), mental health, and social functioning. 

Responses from the general health, physical functioning, role functioning (physical), and bodily pain domains contribute most towards the PCS score, while responses from role functioning (emotional), mental health, and social functioning contributed most towards the creation of the MCS score [25]. Both scores are correlated with vitality, general health (more with the physical score), and social functioning (more with the mental score) [25]. The responses to general health, bodily pain, and mental health were reverse coded so as to align to the direction of the summary score scale. The scores were then combined and normalized to form the corresponding summary score scales using methods described in more detail elsewhere [9]. These summary scores range from 0 to 100, where 0 indicates the lowest level of health and 100 indicates the highest level of health. They were collected at Rounds 2 and 4 of the survey.

#### 2.3.2. Short-Form Six-Dimension 

The Short-Form Six-Dimension (SF−6D) was developed by researchers as a single, preference-based score which can be directly calculated for the SF group (SF−36v2, SF−12v2). These single score measures have applications in economic studies such as cost utility analyses. We calculated this score from the PCS and MCS values, accounting for age and sex [26]. These scores were used to estimate disutility associated with limited functionality and activity, as well as important conditions in the sample of older adults. The utility scores were calculated at Rounds 2 and 4.

#### 2.3.3. Health-Related Quality of Life Comorbidity Index (HRQoL-CI)

A comorbidity index is a weighted measure that helps control for the potential influence of certain illnesses and comorbidities on the outcome of interest [27]. In HRQoL-CI, the illnesses chosen are those that have the greatest impact on health-related quality of life [28]. In this study, we used the index developed by Mukherjee et al. [28]. We used the Clinical Classification Codes provided by the Medical Expenditure Panel Survey to calculate the HRQoL-CI. As per this index, 15 conditions contribute towards MCS and 20 conditions contribute towards PCS. MCS was categorized as scores of 0, 1, 2, 3, and ≥4. PCS was classified as 0, 1–2, 3–4, 5–7, and ≥8. These values were used to validate the concurrent criterion validity of the SF−12v2 instrument and were calculated at Rounds 2 and 4 of the survey. 

#### 2.3.4. Perceived Health and Perceived Mental Health

The perceived health and mental health questions are single item questions that were administered in Rounds 2 and 4 of the survey and asked the respondents to rate their mental and physical health status from poor to excellent. These responses were reverse coded and utilized, while validating the reliability of the SF−12v2 instrument using the test-retest procedure as well as its convergent and discriminant construct validity.

#### 2.3.5. Patient Health Questionnaire—2

The Patient Health Questionnaire—2 (PHQ−2) is an instrument used to screen for depression and scores range from 0–6. The PHQ−2, which includes the first two items on the Patient Health Questionnaire—9 questionnaire, has been previously validated [29]. A higher PHQ−2 score is indicative of a greater tendency for depression. These responses were utilized while validating the convergent and discriminant construct validity of the SF−12v2 instrument. These responses were collected at Rounds 2 and 4 of the survey.

#### 2.3.6. Kessler Scale

The Kessler scale includes six mental health-related questions to assess a person’s non-specific psychological distress during the past 30 days with regards to nervousness, hopelessness, fidgetiness, sadness, effort, and worthlessness [30]. The responses range from “none of the time” to “all of the time”. Higher Kessler scores are indicative of a greater tendency towards mental disability. These responses were utilized while validating the convergent and discriminant construct validity of the SF−12v2 instrument. This was administered at Rounds 2 and 4 of the survey.

#### 2.3.7. Social and Cognitive Limitations 

Social limitations were assessed based on the response to question HE22 (Health Status section) of the survey, which asks about limitations “in participating in social, recreational, or family activities because of an impairment or a physical or mental health problem”. Cognitive limitations were assessed based on responses to the three-part question HE24−01 to HE24−03 (Health Status section) of the survey, which asks whether the individual has experienced “confusion or memory loss”, “problems making decisions” or “requires supervision for their own safety”. These responses were used to validate the predictive criterion validity of the SF−12v2 instrument.

#### 2.3.8. Functional and Activity Limitations

Limitations with regards to work, household or school, as determined by answers to questions HE19 and HE20 (Health Status section) were utilized to assess activity limitations. Functional limitations were determined based on the response to question HE09 (Health Status section) of the survey (“Does anyone in the family have difficulties walking, climbing stairs, grasping objects, reaching overhead, lifting, bending or stooping, or standing for long periods of time?”). These responses were used to validate the predictive criterion validity of the SF−12v2 instrument. The disutility associated with these limitations in older adults was also calculated.

### 2.4. Statistical Analyses 

#### 2.4.1. Reliability 

Reliability is the extent to which a measure is free from random error. Internal consistency is the extent to which all items on a test measure the same thing [31]. The general health, physical functioning, role functioning (physical), and bodily pain domains were tested for correlation with the PCS score of the SF−12v2, while responses from the vitality, role functioning (emotional), role functioning (physical), mental health, and social functioning were tested for correlation with the MCS score of the SF−12v2. Internal consistency of the test was estimated using the Cronbach’s alpha [32].

Test-retest reliability helps understand the degree of stability in a respondent’s answers over time. It is measured using the intraclass correlation coefficient. In short, if the between person variation in response was much more than the within person variation in response (over the two survey administrations in Rounds 2 and 4) then the instrument was considered reliable over the period between the test and the retest period [33]. The test-retest reliability over these two administrations of the questionnaire was only evaluated among those respondents who had identical perceived mental health and perceived health in the corresponding rounds [16].

#### 2.4.2. Validity 

Validity of an instrument is the extent to which it measures what it claims to measure [34]. Reliability is a prerequisite for an instrument to be valid, but the inverse is not true and an instrument can be reliable without being valid [34]. 

Construct validity refers to the degree of logical relationships between related scales or between scales and known disease/patient traits or characteristics [35,36]. To examine the construct validity, we considered the convergent and discriminant validity and carried out confirmatory factor analysis. We tested for convergent and discriminant validity against the PHQ−2 scores, Kessler Index scores, and questions on perceived health and perceived mental health from the same round as MCS/PCS, using the Spearman rank correlation coefficients [18]. Coefficients with values less than 0.3 were considered poor, from 0.3 to 0.5 were considered fair, greater than 0.5 (up to 0.8) were considered moderately strong, and greater than 0.8 were considered very strong [37].

Confirmatory factor analysis is used to assess the fit between observed results and a conceptualized, theoretical model that hypothesizes causal relationship between latent factors and observed indicator variables and test the structural validity of the instrument [38]. We performed confirmatory factor analysis using a two-factor model (PCS and MCS) and tested various goodness of fit indicators [17]. The physical functioning, role functioning (physical), and bodily pain domains were theorized to load on the PCS score, while responses from the role functioning (emotional), mental health, and social functioning were theorized to load on the MCS score [8]. The domains of general health and vitality were theorized to load on both the summary scores and were hypothesized to be correlated [39]. The goodness of fit indicators we reported were: goodness of fit index, adjusted goodness of fit index, root mean square error of approximation, normed fit index, and comparative fit index. The recommended cut off values for the goodness of fit index and adjusted goodness of fit index (greater than/equal to 0.90), normed fit index, and comparative fit index (greater than 0.90), and root mean square error of approximation (<0.05 indicative of a close fit and <0.11 being indicative of an acceptable fit) were compared with the estimated values [17,40,41].

Criterion validity is a measure of the extent to which scores on an instrument correlate to an external, non-test criterion [42]. There are two components to criterion validity. One is concurrent validity, where both the scores from the instrument and the criterion value are measured at the same time [43]. The other is predictive validity, where the criterion value is measured after the scores from the instrument are measured [43]. To examine the concurrent validity of the instrument the PCS and MCS scores were compared against the corresponding scores on the HRQoL-CI. This was done using the one-way analysis of variance test (ANOVA). In addition, the ability of the PCS and MCS instruments to distinguish between those who had HRQoL-CI scores of 0 or ≥1 was determined using the Tukey test. To examine the predictive criterion validity, logistic regression was used where the outcome of interest was limitations in Round 3 and the predictor variable was the summary score (MCS and PCS separately) from the SF−12v2. PCS (Round 2) was set as the predictor variable for functional limitations and activity limitations (Round 3). MCS (Round 2) was the predictor variable for social limitations and cognitive limitations (Round 3).

#### 2.4.3. Disutility 

The disutility related to functional and activity limitations, and important conditions among the older adult sample was calculated by computing the difference in utility between those who had these prior limitations or condition and those who did not. The utility value was determined using the SF−6D utility scores, which were derived from the SF−12v2, adjusting for sex and age [26]. These disutility values were used to test the responsiveness of the scores to limitations and conditions that are important to this group. As these values were derived from the PCS and MCS scores, they too were collected in Rounds 2 and 4 of the survey.

All statistical analyses were performed using SAS (version 9.4 SAS Institute Inc., Cary, NC, US) [44] and R programming software [45]. An overview of the methods is depicted in Table A1.

## 3. Results

### 3.1. Demographic Information

The demographic characteristics of the sample are depicted in Table 1. The final sample consisted of 1040 individuals (Figure 1). The sample was predominantly white (68.3%), female (57.2%), and from the south (41.3%). While the majority was currently married (50.8%), a large proportion was either widowed, divorced, or separated (43.6%). Most of the people were on Medicare, with nearly half of the Medicare beneficiaries also having private insurance. The majority of the sample had no limitation in work/school/household activity (80.2%) and physical functioning (64.4%). The average age was 74.09 years (standard deviation (SD): 6.19). The mean PCS and MCS scores were 41.89 (SD: 12.11) and 53.10 (SD: 9.30), respectively. Also, the sample had mean HRQoL-CI of 5.00 (SD: 3.51) and 1.40 (SD: 1.68) for physical and mental comorbidities, respectively. The mean SF−6D utility score for the people in the study sample was 0.77 (SD: 0.14).

### 3.2. Reliability

A Cronbach alpha score of 0.7 or greater is considered indicative of acceptable internal consistency [32,46]. PCS had a Cronbach alpha value of 0.87 and MCS had a Cronbach alpha value of 0.86. These indicate a high degree of internal consistency. PCS had an intraclass correlation coefficient score of 0.79, while the intraclass correlation coefficient score for MCS was 0.59. These results are indicative of PCS having good reliability and MCS having moderate test-retest reliability [47].

### 3.3. Validity

The results of the convergent and divergent construct validity for PCS and MCS are depicted in Table 2. While the question on perceived mental health had a fair relationship with both MCS (r = 0.37) and PCS (r = 0.35), it had a stronger association with MCS. The question on perceived health had a moderately strong relationship with PCS (r = 0.58) and fair relationship with MCS (r = 0.31). The PHQ−2 (r = −0.59) and Kessler Index (r = −0.66) scores had moderately strong associations with MCS. The PHQ−2 (r = −0.33) and Kessler Index (r = −0.39) scores had a fair relationship with PCS. MCS and PCS were poorly related with each other (r = 0.12).

**Figure 2:**
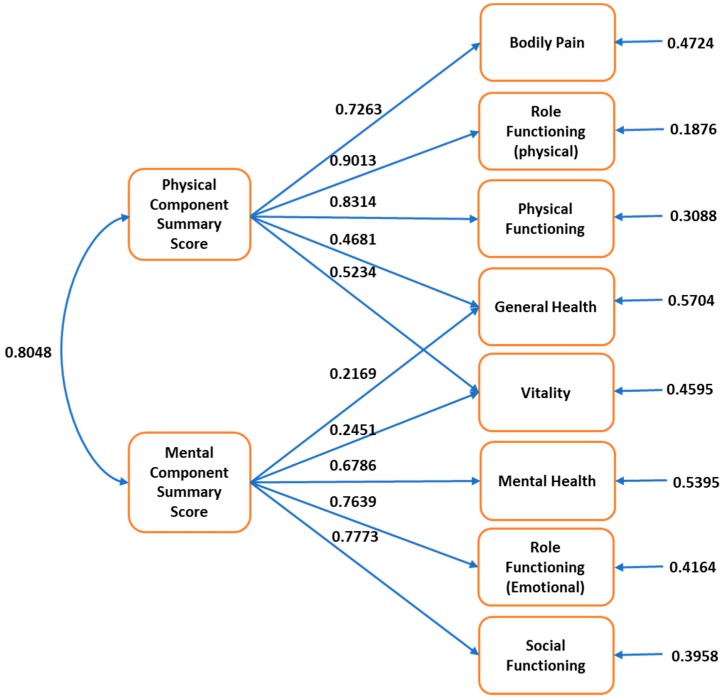
Results of confirmatory factor analysis for structural validity of Physical Component Summary Score and Mental Component Summary Score in the Short-Form 12-Item Survey—version 2 among an older (65 years or greater) US population.

The results of the confirmatory factor analysis are depicted in Figure 2 and Table 3. The goodness of fit index was 0.9588, the adjusted goodness of fit index was 0.9128, the root mean square error of approximation was 0.1004, the normed fit index was 0.9578, and the comparative fit index was 0.9596. These values were adequate, and the observed model showed good fit with the theoretical model.

**Figure 3:**
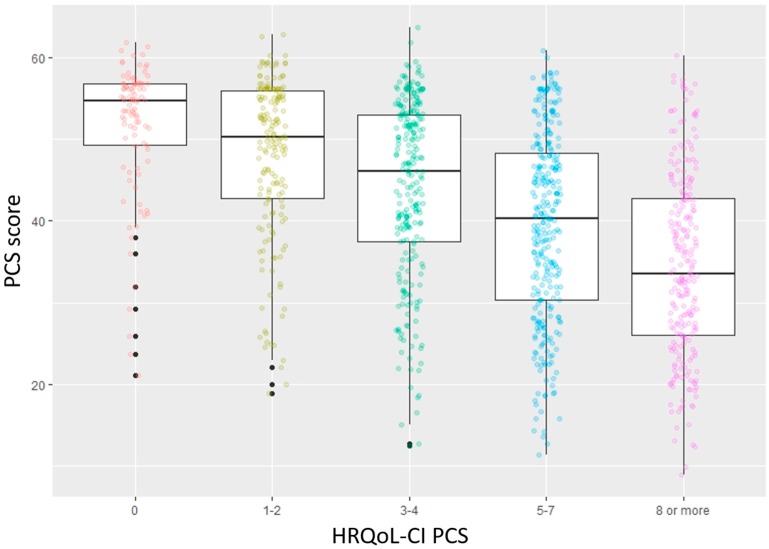
Results for concurrent criterion validity of Physical Component Summary Score in the Short-Form 12-Item Survey—version 2 among an older (65 years or greater) US population (PCS score: Physical Component Summary Score; HRQoL-CI PCS: Health-Related Quality of Life Comorbidity Index (Physical Component Score)).

**Figure 4:**
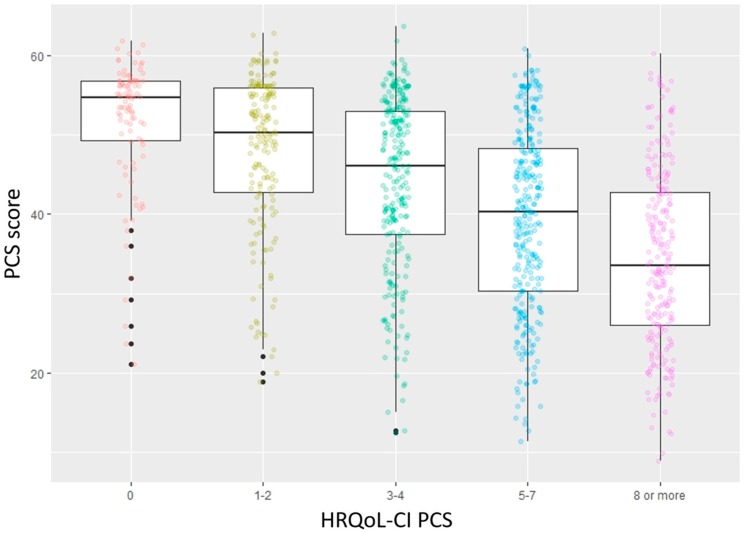
Results for concurrent criterion validity of Mental Component Summary Score in the Short-Form 12-Item Survey—version 2 among an older (65 years or greater) US population (MCS score: Mental Component Summary Score; HRQoL-CI MCS: Health-Related Quality of Life Comorbidity Index (Mental Component Score)).

The results of the concurrent criterion validity for PCS and MCS are illustrated in Figure 3 and Figure 4, respectively. There was a statistically significant decrease in PCS and MCS as the corresponding comorbidity scores increased. This change was also significant for both instruments, between those with corresponding HRQoL-CI scores of 0 and greater than 0. 

A 1-unit increase in the Round 2 MCS score was associated with decreased odds of future social limitations (odds ratio (OR): 0.948; 95% confidence interval (CI): 0.930, 0.965) and cognitive limitations (OR: 0.920; 95% CI: 0.903, 0.937) in Round 3. Correspondingly, a 1-unit increase in the round 2 PCS score was associated with decreased odds of future activity limitations (OR: 0.885; CI: 0.870, 0.900) and functional limitations (OR: 0.877; CI: 0.863, 0.891) in Round 3. All these values were statistically significant at the 95% level. 

### 3.4. Disutility

Table 4 depicts the disutility associated with functional and activity limitations, as well as relevant and important medical conditions among those aged greater than or equal to 65 years, using the SF−6D scale. Activity limitations were associated with a disutility of 0.18 and functional limitations were associated with a disutility of 0.15. Arthritis, coronary heart disease, diabetes, myocardial infarction, stroke, angina, and high blood pressure were associated with a disutility of 0.06, 0.07, 0.07, 0.06, 0.09, 0.06, and 0.08 respectively. These values are indicative of the responsiveness of the instrument to limitations and conditions that are of importance to older adults. 

## 4. Discussion

Health-related quality of life in older adults has become increasingly important, especially as the population ages. Previous studies have used the SF−12v2 instrument to quantify health-related quality of life in an older population [11,12,13,14]. However, to the best of our knowledge, no previous study has assessed the validity and reliability of the SF−12 in an older US population. 

This study found that both PCS and MCS demonstrated acceptable internal consistency and good and moderate test-retest reliability. For test-retest reliability testing, the interval between the tests is important. It should be long enough that carryover effects (due to memory, practice, or mood) are not a problem, but short enough that a change in status has not occurred [48]. We had a longer period but ensured that there was not a change in status by requiring that the participants had unchanged perceived mental and perceived health in this period, using methods similar to those used by Cheak-Zamora et al. [16]. We found that PCS has good test-retest reliability, while MCS has moderate test-retest reliability.

Perceived mental health had a fair relationship with both MCS and PCS, with a slightly stronger association with MCS. Perceived health had a strong association with PCS and a fair association with MCS. The Patient Health Questionnaire—2 and Kessler Index scores had strong associations with MCS and a fair relationship with PCS. MCS and PCS were very poorly related with each other. These findings were as expected and similar to those found by previous studies that validated the SF−12v2 using Medical Expenditure Panel Survey data, albeit in different populations [16,18,19]. Thus, the questionnaire demonstrated sufficient convergent and discriminant ability. Confirmatory factor analysis, for both MCS and PCS, showed adequate fit with the theoretical model. Both MCS and PCS also demonstrated concurrent criterion validity, as well as predictive criterion validity. Thus, PCS and MCS should be able to predict future limitations in physical and mental health. The disutility measures highlight the significant impact that limitation in activity and functioning, and important conditions in this population, have on the quality of life.

A previous study that assessed the reliability and validity of the SF−12v2 instrument in an elderly Chinese population (Xujiahui district of Shanghai) found that the SF−12 was a reliable and valid instrument for this population [49]. However, they were not able to assess test-retest validity of the instrument. Another study in Sweden failed to demonstrate construct validity of the SF−12 in the general elderly Swedish population [50]. However, there were group differences between those that did answer the survey and those that did not, as well as missing data. Also, the sample was that of those greater than or 75 years of age. Using Medical Expenditure Panel Survey data, another study found that PCS scores correlated with healthcare costs and utilization in older adults, but that study did not assess MCS or consider the reliability and validity of these scores over time [15].

There were some limitations to this study. The data source was from a survey, and consequently the sample was subject to survey and recall bias. In the calculation of disutility, adjustment for comorbidities was not taken into consideration. Furthermore, it would have been useful to have been able to compare the results with those of the EQ−5D, however, this measure is no longer available in the Medical Expenditure Panel Survey data. Also, we did not have information on institutionalized individuals (e.g., nursing homes), and this may affect the generalizability of the results to only community-dwelling older adults. While our cohort was approximately one-third non-white race, replication across racial groups is needed in future research. Further assessing the predictive ability of the SF−12v2 with additional measures is also needed. However, despite these limitations, the results of this study help increase confidence in the utilization of health-related quality of life measures in this population, which will hopefully lead to a greater importance being given to this domain of health among older adults. 

## 5. Conclusions

This study provides evidence that demonstrates the validity and reliability of the SF−12v2 instrument in an older population, and hence this health-related quality of life measure should be used in this population to measure these outcomes.

## Figures and Tables

**Table 1:** Demographic characteristics of sample at baseline.

Variable	Frequency (Total: 1040)	Percent (%)/Standard Deviation
**Race**		**Percentage**
White	710	68.3%
Black	201	19.3%
Other	129	12.4%
**Region in round 1**		
Northeast	148	14.2%
Midwest	205	19.7%
South	429	41.3%
West	258	24.8%
**Marital status at round 1**		
Never Married	58	5.6%
Widowed/divorced/separated	453	43.6%
Currently married	529	50.8%
**Insurance coverage for baseline year**		
Medicare only	389	37.4%
Medicare and private	468	45.0%
Medicare and other public	174	16.7%
Uninsured	6	0.6%
No Medicare and any public/private	3	0.3%
**Sex**		
Female	595	57.2%
Male	445	42.8%
**Limitation in work/housework/school activities at round 1**		
Yes	206	19.8%
No	834	80.2%
**Limitation in physical functioning at round 1**		
Yes	370	35.6%
No	670	64.4%
**Continuous Variables**	**Mean**	**Standard deviation**
Age at round 1 (years)	74.09	6.19
PCS score ^a^	41.90	12.11
MCS score ^b^	53.10	9.30
Short-Form Six-Dimension (SF−6D) score	0.77	0.14
HRQoL-CI-PCS score ^a^	5.00	3.51
HRQoL-CI- MCS score ^b^	1.40	1.68

^a^ PCS score: Physical Component Summary score, ^b^ MCS score: Mental Component Summary score.

**Table 2:** Spearman rank correlation coefficients for construct (convergent and discriminant) validity of Physical Component Summary Score and Mental Component Summary Score in the Short-Form 12-Item Survey—version2 among an older (65 years or greater) US population ^1^.

Measure	Perceived Mental Health	Perceived Health	Patient Health Questionnaire—2	Kessler Scale	Mental Component Summary Score
Physical Component Summary Score	0.35	0.58	−0.33	−0.39	0.12
Mental Component Summary Score	0.37	0.31	−0.59	−0.66	-

^1^ The Spearman rank correlation coefficients were classified into poor (less than 0.3), fair (0.3 to 0.5), moderately strong (greater than 0.5 to 0.8), and very strong (greater than 0.8).

**Table 3:** Fit summary statistics of confirmatory factor analysis for structural validity of Physical Component Summary Score and Mental Component Summary Score in the Short-Form 12-Item Survey—version 2 among an older (65 years or greater) US population ^1^.

Measure	Value
Goodness of Fit Index	0.9588
Adjusted Goodness of Fit Index	0.9128
Root Means Square Error of Approximation	0.1004
Bentler Comparative Fit Index	0.9596
Bentler–Bonett Normed Fit Index	0.9578

^1^ The recommended cut-off values for the goodness of fit index and adjusted goodness of fit index (greater than/equal to 0.90), normed fit index and comparative fit index (greater than 0.90), and root means square error of approximation (<0.05 indicative of a close fit and <0.11 being indicative of an acceptable fit).

**Table 4:** Disutility values from the derived Short-Form Six-Dimension (SF−6D) instrument.

Subpopulation	Yes	No	Disutility
Activity limitation	0.62	0.80	0.18
Functional limitation	0.67	0.82	0.15
Arthritis	0.80	0.74	0.06
Coronary Heart Disease	0.78	0.71	0.07
Diabetes	0.78	0.71	0.07
Myocardial infarction	0.77	0.71	0.06
Stroke	0.78	0.69	0.09
Angina	0.77	0.71	0.06
High Blood Pressure	0.82	0.74	0.08

## Appendix A

**Table A1:** Summary of methods used in the testing of the reliability and validity of Physical Component Summary Score and Mental Component Summary Score in Short Form 12-Item Survey version−2 among an older (65 years or greater) US population.

Type	Subtype	Measures Used
**Reliability**	Internal consistency	Cronbach alpha
	Test-retest	Intra-class correlation
**Validity**		
Construct Validity	Convergent and discriminant	Spearman rank correlation
	Structural	Confirmatory factor analysis
Criterion Validity	Concurrent	Analysis of variance, Tukey test
	Predictive	Logistic regression
